# A copper-dependent compound restores ampicillin sensitivity in multidrug-resistant *Staphylococcus aureus*

**DOI:** 10.1038/s41598-020-65978-y

**Published:** 2020-06-02

**Authors:** Cameron L. Crawford, Alex G. Dalecki, Mildred D. Perez, Kaitlyn Schaaf, Frank Wolschendorf, Olaf Kutsch

**Affiliations:** Department of Medicine, University of Alabama at Birmingham, Birmingham, AL USA

**Keywords:** Antimicrobials, Bacteria, Pathogens

## Abstract

Multi-drug resistant *Staphylococcus aureus*, including methicillin-resistant *S. aureus* (MRSA), has become a worldwide, major health care problem. While initially restricted to clinical settings, drug resistant *S. aureus* is now one of the key causative agents of community-acquired infections. We have previously demonstrated that copper dependent inhibitors (CDIs), a class of antibiotics that are only active in the presence of copper ions, are effective bactericidal agents against MRSA. A second-generation CDI, APT-6K, exerted bactericidal activity at nanomolar concentrations. At sub-bactericidal concentrations, it effectively synergized with ampicillin to reverse drug resistance in multiple MRSA strains. APT-6K had a favorable therapeutic index when tested on eukaryotic cells (TI: > 30) and, unlike some previously reported CDIs, did not affect mitochondrial activity. These results further establish inhibitors that are activated by the binding of transition metal ions as a promising class of antibiotics, and for the first time, describe their ability to reverse existing drug resistance against clinically relevant antibiotics.

## Introduction

The rapid increase of antibiotic resistance within bacterial populations is associated with longer hospital stays, increased treatment costs, and more patient deaths^1,2^. An estimated 700,000 individuals die each year as a result of infections with antibiotic resistant bacteria, and the amount of deaths are expected to increase if no alternative, effective therapies are developed^3^. New antibiotics are essential to avoid a public health crisis. The identification of new antibiotics for *Staphylococcus aureus* is an especially urgent task, with antibiotic resistance in this bacterium already observed against some of the last line of defense antibiotics such as vancomycin, linezolid, and daptomycin^4^. An alternative to the discovery of new antibiotics are drugs that restore the efficacy of available antibiotics and overcome bacterial drug resistance mechanisms. Ideal drugs would be ones that are both effective by themselves and that restore the activity of current antibiotics by reversing antibiotic resistance^5,6^.

Copper dependent inhibitors (CDIs) are a functionally new type of antibiotic gaining increased appreciation due to their ability to inhibit drug resistant bacteria such as *S. aureus, Mycobacterium tuberculosis, Mycoplasma spp*., and *Neisseria gonorrheae*^7–13^. These compounds utilize copper for their activities and include the FDA approved drug disulfiram and anti-cancer compounds like 8-hydroxyquinoline (8HQ)^7,9,13–15^. Hundreds of new CDIs with antibacterial and antifungal activity have been identified in drug screens against *S. aureus, M. tuberculosis*, and *Cryptococcus neoformans* using defined culture medium that contains physiologically relevant concentrations of copper that were previously not identified in these compound libraries when screened under industrial standard conditions (no consideration of transition metal concentrations), demonstrating the untapped potential of CDIs^10,12,15,16^.

At present, it is unclear whether CDIs target a shared bacterial pathway or whether they target a large array of different functionalities. Some investigations have shown that CDIs have the ability to shut down different ATP generating processes such as oxidative phosphorylation and glycolysis^11,17^. Studies by others have shown that inhibition of ATP generation can restore sensitivity to different antibiotics in drug resistant bacteria. Examples of this phenomenon include increasing the sensitivity of *S. aureus* to polymyxins with the ATP synthase inhibitor oligomycin A or improving the efficacy of β-lactam antibiotics against *Mycobacterium tuberculosis* with the electron transport chain inhibitors 2-aminoimidazoles (2-AIs)^18,19^. Given reports that some CDIs affect ATP generation, we tested a second-generation CDI called APT-6K and found that it has rapid bactericidal activity in the presence of copper and greatly reduces ATP concentrations prior to cell death. We demonstrate the ability of APT-6K to overcome pre-existing drug resistance in *S. aureus* and that APT-6K, at concentrations that exert no anti-bacterial effect, restored the activity of ampicillin in resistant MRSA isolates.

## Results and Discussion

### APT-6K is a potent copper-dependent inhibitor of *S. aureus*

**Figure 1:**
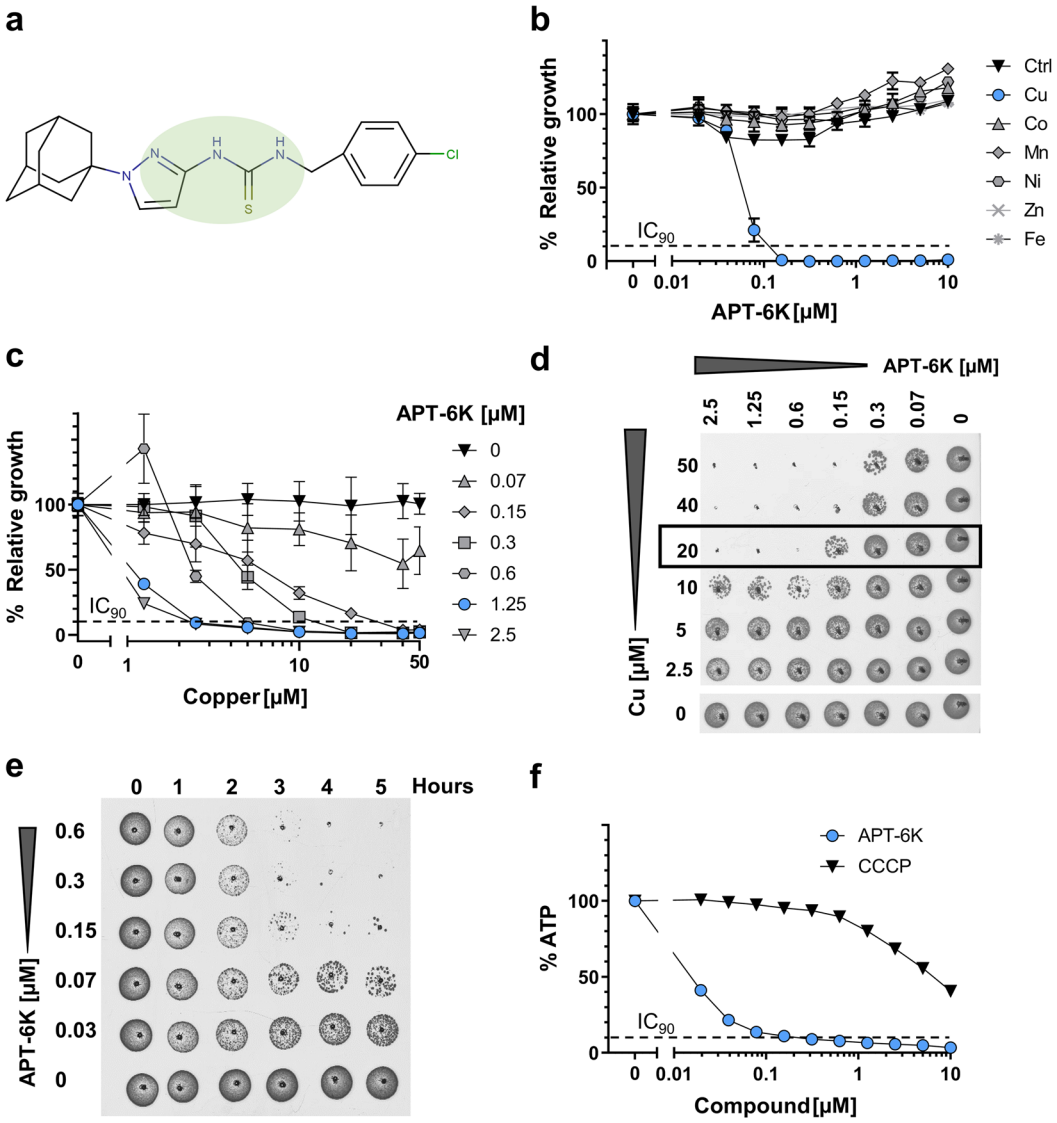
APT-6K is a copper specific bactericide that rapidly reduces ATP levels. (**a**) Structure of APT-6K. The green circle denotes the NNSN motif. (**b**) Activity of APT-6K against *S. aureus* strain Newman in media supplemented with 50 µM copper (Cu) (blue circles), cobalt (Co), manganese (Mn), nickel (Ni), zinc (Zn), or iron (Fe). (**c**) APT-6K and Cu were titrated against each other in a microplate assay and bacterial growth of the *S. aureus* strain Newman was determined after 20 hours. (**d**) Culture samples from each condition of the microplate assay in (d) were transferred into drop assays to determine whether any observed growth inhibition in (d) had been bacteriostatic (outgrowth) or bactericidal (no outgrowth) in nature. Results for inactive copper concentrations below 2.5 µM were removed from the presentation. (**e**) Time to kill kinetics of *S. aureus* strain Newman exposed to increasing concentrations of APT-6K in the presence of 50 µM Cu. (**f**) ATP levels in *S. aureus* strain Newman after exposure for one hour to APT-6K + 50 µM Cu or CCCP, a mitochondrial oxidative phosphorylation uncoupler. All values were normalized to the untreated controls of each series.

A previous compound screen identified a group of antibiotics that exerted potent anti *S. aureus* activity and was characterized by a nitrogen-nitrogen-sulfur-nitrogen (NNSN) motif forming the structural backbone (Fig. 1a, green circle). These compounds only exhibited antibiotic activity in the presence, but not the absence, of copper^10^. A sub-group of NNSN compounds, which we described as adamantyl-bearing pyrazolyl-thioureas (APT), were further investigated for their activity against *S. aureus*. These were of particular interest as adamantyl-groups have been reported to convey stability to compounds, a desirable feature for antibiotics^20^. The inhibitor APT-6K (Fig. 1a) is a second generation copper dependent APT with a minimum inhibitory concentration (MIC) of 150 nM on *S. aureus* strain Newman in the presence of 50 µM copper, the transition metal concentration that was used in the drug screen (Fig. 1b, blue circles). Of note, copper concentrations in serum range between 10–20 µM and can reach 400 µM within phagolysosomes, where copper ions are part of a physiological anti-bacterial defense mechanisms, but in our experiments 50 µM copper alone is not growth inhibitory^21–23^. The inhibitor APT-6K was found to be highly copper-specific in its anti-bacterial activity, as no other transition metals would activate the compound (Fig. 1b).

To determine the minimal copper concentration required for APT-6K to exert an anti-bacterial activity, copper and APT-6K were titrated against each other (microplate assay) in suspension cultures and following overnight incubation, were transferred to nutrient rich agar plates. No measurable bacterial growth in liquid culture was observed starting at APT-6K concentrations of 1.25 µM or higher in combination with copper concentrations as low as 2.5 µM (Fig. 1c; blue circles). Transfer of these cultures to agar plates enabled bacterial outgrowth, indicating that the effect had been bacteriostatic (Fig. 1d). Bactericidal activity required a minimum copper concentration of 20 µM but was achievable at lower APT-6K concentrations (Fig. 1d; black box). For either bacteriostatic or bactericidal effects, the required copper concentrations remained well within the physiologically relevant range.

Time to death experiments were performed where APT-6K was titrated in the presence of 50 µM copper into bacterial cultures to determine the length of time needed for APT-6K to exert its bactericidal activity. Samples were removed at the indicated time points and transferred to nutrient rich agar plates for bacterial outgrowth. A reduction in bacterial viability could be observed as early as two hours following APT-6K addition, and after five hours of treatment with 300 nM APT-6K, no viable bacteria could be recovered (Fig. 1e). In addition, there was an observable reduction in bacteria at APT-6K concentrations as low as 30 nM.

Previous work had shown that some CDIs can affect different ATP generating processes^11,17,24^. To understand whether APT-6K would act through this mechanism, we measured intracellular ATP concentrations after APT-6K treatment in the presence of 50 µM copper. ATP-6K was indeed extremely potent at interfering with ATP generation. After one hour of treatment with carbonyl cyanide m-chlorophenyl hydrazone (CCCP; 10 µM), an established inhibitor of oxidative phosphorylation in *S. aureus* that is frequently used as a positive control, ATP levels had dropped to 40% of the untreated control^25^. During the same period, treatment with as little as 300 nM APT-6K reduced ATP concentrations to <10% when compared to the ATP concentration in untreated bacteria (Fig. 1f), suggesting that interference with ATP generation is contributing to the antibiotic effect of APT-6K.

### APT-6K is well tolerated by eukaryotic cells

**Figure 2:**
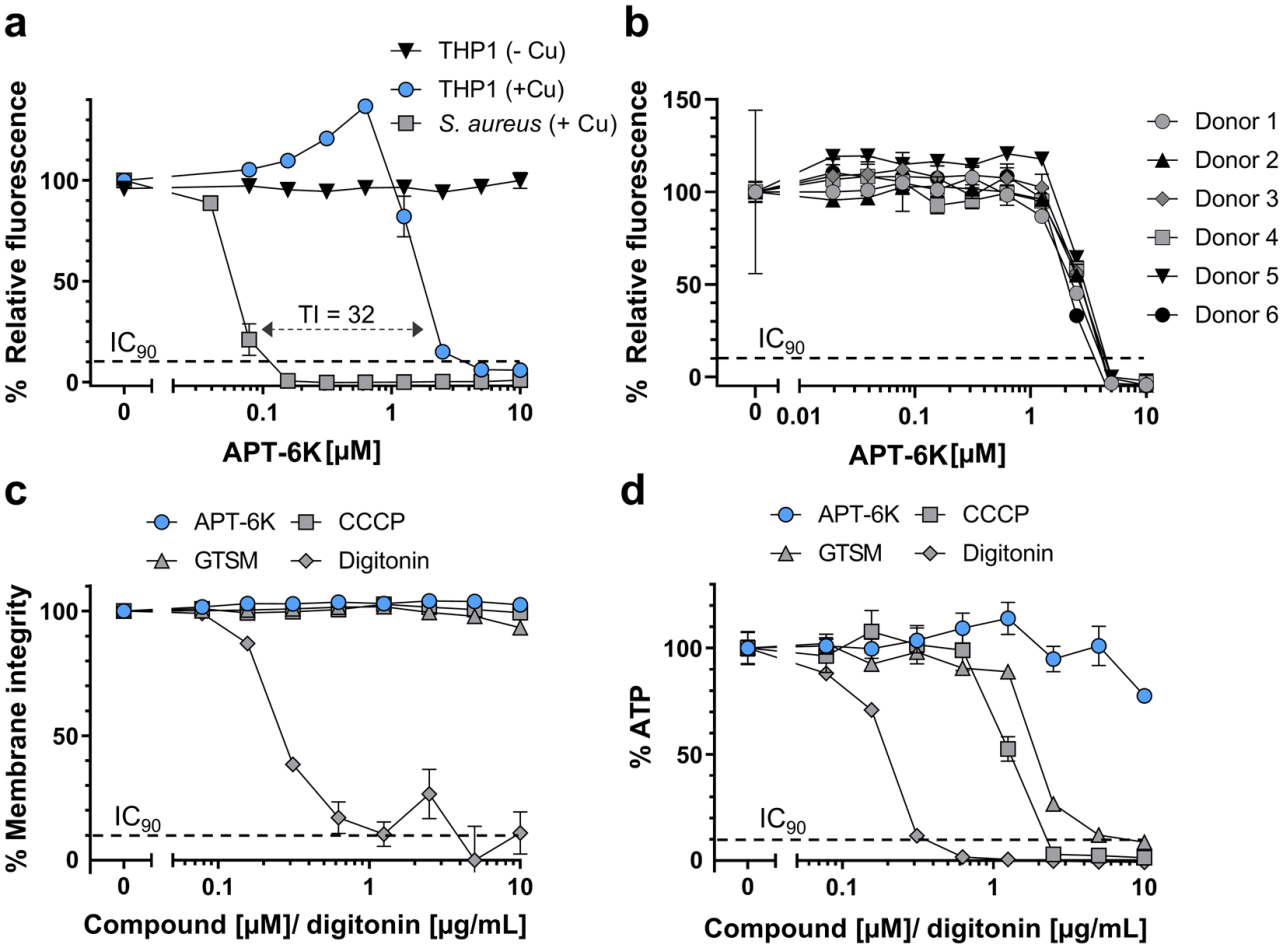
Eukaryotic toxicity profile of APT-6K. (**a**) THP-1 cells were treated with APT-6K, with and without 50 µM copper added to the medium. *S. aureus* inhibition is shown side by side to illustrate an acceptable therapeutic index (TI) of 32. (**b**) PMBCs from six donors were treated with APT-6K and 50 µM copper. (**c**) Effects of digitonin, GTSM, APT-6K, and CCCP on THP-1 membrane integrity by measuring dead cell protease activity. Copper was included at 50 µM for APT-6K and GTSM. (**d**) Inhibition of respiratory ATP synthesis upon challenge with digitonin, CCCP (a positive control for mitochondrial toxicity), GTSM (a known mitochondrial inhibitor) and APT-6K. GTSM and APT-6K were assayed in the presence of 50 µM copper. Concentrations were in micromolar for APT-6K, CCCP, and GTSM and microgram/mL for digitonin. All normalized values have been normalized to the untreated controls of each series.

An essential requirement for any compound to be considered as a potential antibiotic lead is a relevant therapeutic index indicating the absence of toxicity against eukaryotic cells at concentrations that exert antibacterial activity. To determine APT-6K toxicity against human cells, APT-6K in the absence and presence of copper was titrated on THP-1 cells, a human monocytic cell line, and metabolic activity as a surrogate for cell viability was determined 24 hours post treatment using resazurin. By itself, APT-6K exerted no toxicity up to the maximum tested concentration of 10 µM. In the presence of copper, the toxic concentration (TC_90_) was determined to be 5 µM (Fig. 2a). Toxicity experiments using peripheral blood mononuclear cells (PBMCs) from six healthy human donors produced an identical TC_90_ when metabolic activity was used as a readout (Fig. 2b). Flow cytometric analysis of T cells treated with 1.25 µM or less APT-6K plus copper suggested no impairment of protein synthesis based on the expression levels of constitutively expressed T cell markers, such as CD3, CD4, CD8, or CD28 (Supplementary Figures 1–3). Higher concentrations of APT-6K reduced cell surface marker expression in some of the tested donors. APT-6K did not induce the expression of any T cell activation markers (CD25, CD38, CD69, and PD1) or any markers that would indicate pro-inflammatory activity (TNF-α, IFN-γ, and granzyme B) (Supplementary Figures 4 and 5), another important characteristic for lead compounds. In summary, these data suggest an initial therapeutic index of 16–32 relative to the determined MIC_90_ against *S. aureus* (0.15 µM), which is well above the therapeutic index threshold of 10 that is commonly used to consider compounds for initial lead optimization^26^.

Previous work by others has described the eukaryotic toxicity of some CDIs as being, at least in part, due to mitochondrial inhibition. We thus tested for possible effects of APT-6K in the presence of copper on membrane integrity and mitochondrial function as a mechanism of the observed toxicity of APT-6K against eukaryotic cells at high concentrations (>3 µM). For these experiments we used glyoxal-bis(N(4)-methyl-3-thiosemicarbazone (GTSM), a comprehensively characterized copper-dependent antibacterial compound reported to inhibit Complex I within the electron transport chain of both prokaryotes and eukaryotes, as a defined CDI control^24^. CCCP, a mitochondrial oxidative phosphorylation uncoupler^27^, and digitonin, a mild nonionic detergent frequently used to permeabilize cell membranes^28^, served as positive controls for mitochondrial toxicity and membrane integrity effects, respectively. The ToxGlo (Promega) mitochondrial toxicity assay allows for the quantification of both effects. Briefly, a quenched fluorogenic peptide substrate is added (bis-alanyl-alanyl phenylalanyl- rhodamine 110; bis-AAF-R110) that cannot permeate through an intact membrane but following permeation through a compromised cell membrane, it is proteolytically cleaved by a distinct necrosis-associated protease, triggering a fluorescent signal. A second step in the assay determines mitochondrial toxicity by quantifying ATP levels produced under the respective experimental conditions.

As seen in Fig. 2c, 90 minutes after compound addition, membrane integrity was not affected by either GTSM or APT-6K in the presence of copper, or by CCCP. As expected, the membrane integrity was compromised by digitonin, acting as the positive control.

In the absence of any membrane damage, GTSM in the presence of copper and CCCP resulted in a sharp decrease in ATP production in THP-1 cells. In contrast, APT-6K treatment in the presence of copper did not result in any significant loss of ATP production, indicating the absence of mitochondrial toxicity (Fig. 2d). While this does not exclude secondary mitochondrial toxicity effects of APT-6K at later time points, it is an exciting finding as the observed mitochondrial toxicity of GTSM most likely contributes to its reported *in vitro* toxicity^17^. Despite its apparent cell culture toxicity, GTSM is tolerated in mice and has reported anti-cancer and anti-Alzheimer’s activity^29–31^. As such, the results for APT-6K are promising as they suggest that reduced *in vitro* mitochondrial toxicity may translate into improved therapeutic potential.

### Antibiotic effect of APT-6K on multidrug resistant *S. aureus*

**Figure 3:**
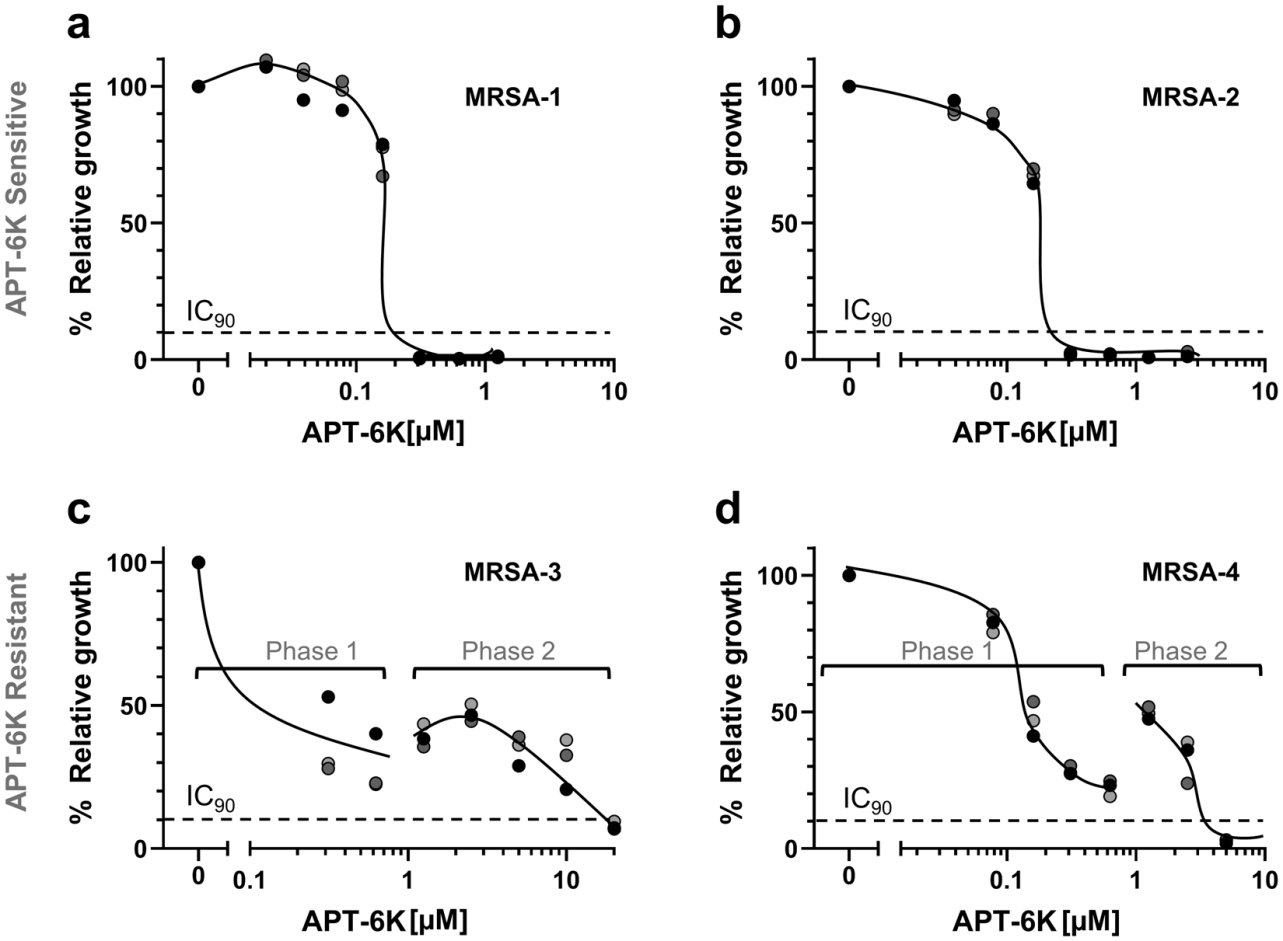
Differential activity of APT-6K against clinical MRSA isolates. The antibacterial effect of APT-6K (+50 µM Cu) was tested against four clinical *S. aureus* MRSA isolates termed MRSA-1 to MRSA-4. (**a**) MRSA-1 and (**b**) MRSA-2 were found to be APT-6K sensitive in a concentration range comparable to a non-drug resistant *S. aureus* laboratory strain. (**c**) MRSA-3 and (**d**) MRSA-4 were found to be APT-6K tolerant, exhibiting a bi-phasic response profile to increasing APT-6K concentrations. In the therapeutically relevant concentration range APT-6K exerted an inhibitory effect but could not suppress bacterial growth below the IC_90_ value (phase 1). In phase 2, additional APT-6K concentration increases initially reversed the inhibitory effect prior to accomplishing complete inhibition at APT-6K concentrations between 5–20 µM. All values were normalized to the untreated controls of each series. The results of three technical replicates are indicated by symbols in different gray shades.

Another criterium for the development of novel antibiotics is their performance against bacterial strains with pre-existing antibiotic resistances. To determine whether the antibiotic activity of APT-6K was maintained against MDR/MRSA strains, we tested a panel of multi-drug resistant clinical *S. aureus* isolates (Supplementary Table S1). While some of these isolates, represented by MRSA-1 and −2, were found to be sensitive to copper-activated APT-6K in a concentration range similar to what was observed for the Newman laboratory strain (MIC = 300 nM) (Fig. 3a,b), we identified two isolates, MRSA-3 and −4, that naturally had an increased tolerance to APT-6K with MIC_90s_ of 20 µM and 5 µM, respectively (Fig. 3c,d). The inhibitory effect of APT-6K on the two sensitive MRSA isolates could be titrated, but the response to APT-6K on the resistant strains was bi-phasic. We observed an initial inhibitory effect at low concentrations (<1 µM) that never suppressed bacterial growth below the MIC_90_ cut-off (Phase 1). Additional increases in APT-6K concentrations rendered APT-6K initially less effective (Fig. 3c,d), before the second onset of APT-6K antibacterial activity at even higher concentrations (Phase 2). It is important to emphasize that APT-6K at concentrations below 1 µM still reduced viability of these MRSA isolates by ~75%.

For other CDIs, we have previously measured a small increase in the effective concentration in some MRSA strains, but this is the first time we observed pre-existing, natural tolerance against a CDI in select MRSA strains. Recent publications have highlighted two previously unknown copper resistant proteins in a MRSA isolate (USA-300/JE2) termed CopX and CopL, in addition to the already reported copper resistance pump CopA and the copper chaperone CopZ^32,33^. CopA and CopZ are found in every *S. aureus* strain, while the presence of CopX and CopL seems to be limited to MRSA isolates^32,34^, making them candidate genes for the observed APT-6K tolerance phenotype of MRSA-3 and MRSA-4.

**Figure 4:**
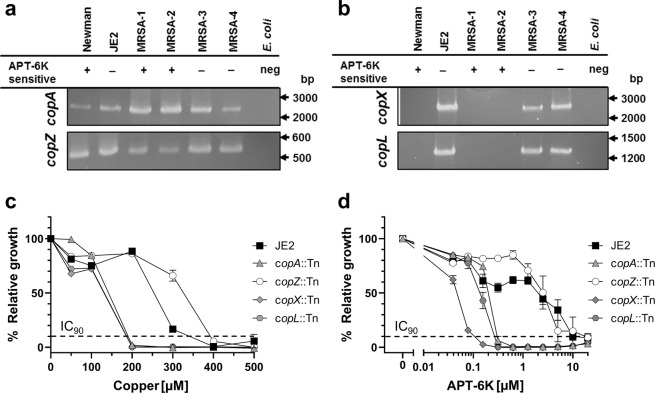
Copper resistance affects APT-6K sensitivity in *S. aureus*. Primers for the full length genes of (**a**) *copA* and *copZ* or (**b**) *copX* and *copL* were used in colony PCR to amplify the target genes from Newman, JE2, MRSA-1, MRSA-2, MRSA-3, MRSA-4, and *E. coli* (negative control). (**c**) JE2 and JE2 with transposons in *copA, copZ, copX*, or *copL* were treated with dilutions of copper. (**d**) JE2 and JE2 with transposons in *copA, copZ, copX*, or *copL* were treated with dilutions of APT-6K in the presence of 50 µM copper. The expected band sizes were 2791 bp for *copA*, 566 bp for *copZ*, 2729 bp for *copX*, and 1263 bp for *copL*. The gels have been cropped for clarity, please see Supplementary Figures 6 and 7 for the full gels. All normalized values have been normalized to the untreated controls of each series.

We first tested the four clinical MRSA strains, USA-300/JE2 (the MRSA strain used to describe *copX* and *copL)*, and the laboratory Newman strain for the presence of *copA*, *copZ*, *copX* and *copL* genes. PCR revealed that, as expected, all strains/isolates had the *copA* and *copZ* locus (Fig. 4a and Supplementary Figure 6). However, Newman and the APT-6K sensitive MRSA-1 and MRSA-2 isolates had neither the *copX* nor the *copL* genes. In contrast, USA300/JE2, as reported by others^32,34^, and the two APT-6K tolerant MRSA isolates had the *copX* and *copL* genes, suggesting a possible correlation of APT-6K resistance with the presence of CopX and/or CopL proteins (Fig. 4b and Supplementary Figure 7). Copper titrations on the different *S. aureus* strains/isolates tracked with the presence of *copX* and *copL*. While MRSA-1 and MRSA-2 were only copper tolerant up to a concentration of 125 µM, USA300/JE2 and the *copX/copL*-positive MRSA-3 and MRSA-4 were copper resistant with a MIC of 500 µM (Supplementary Figures 8).

This prompted us to specifically test the effects of the various copper resistant genes in the JE2 MRSA strain, for which transposon mutants with inactivated *copA*, *copZ*, *copX* or *copL* are available. As seen in Fig. 4c, individual inactivation of *copA, copX, or copL* all reduced copper tolerance relative to the parental USA-300/JE2 strain, suggesting that these proteins provide non-redundant activities. Interestingly, inactivation of *copZ* had no effect on copper tolerance in this experimental system. We next tested the sensitivity of the USA-300/JE2 strains to APT-6K as a function of the presence of *copA*, *copZ*, *copX* or *copL*. As expected, the parent USA-300/JE2 cells, had a MIC similar to that of the two APT-6K tolerant MRSA isolates (Fig. 4d). Transposon-induced inactivation of c*opA, copX*, or c*opL* rendered JE2 cells APT-6K sensitive, similar to MRSA-1 and MRSA-2 (~60–100-fold reduction in APT-6K tolerance). However, *copZ* inactivation had no effect on APT-6K tolerance. These data link APT-6K tolerance of selected MRSA to the presence of the recently discovered *copX* and *copL* genes.

Given the abundance of literature describing how the occurrence of metal-resistance, including copper-resistance, in bacteria is associated with the occurrence of antibiotic drug resistance, we speculated that the APT-6K tolerance mechanism may overlap with other antibiotic resistance mechanisms^35–38^. We thus decided to test whether APT-6K, when co-administered with other antibiotics, could overcome bacterial resistance mechanisms and restore antibiotic sensitivity.

### APT-6K restores ampicillin activity against of MRSA isolates

A particularly interesting antibiotic for these experiments is ampicillin, as recent studies have provided proof of concept that β-lactam resistance in *S. aureus* can be reversed. For example, daptomycin has been demonstrated to restore β-lactam sensitivity in MRSA strains, and vice versa, β-lactams restored daptomycin sensitivity^39,40^. More importantly, ampicillin is on the World Health Organization’s Model List of Essential Medicines due to its low toxicity, low cost of production, and efficaciousness^41^. However, resistance to methicillin, which implies ampicillin resistance, is widespread throughout the world with over 50% of *S. aureus* clinical isolates in the United States and parts of Europe and Asia testing positive for methicillin resistance^42^.

To test whether ampicillin would regain antibacterial activity in the presence of APT-6K, we first established the inhibitory concentrations of ampicillin and APT-6K in the presence of copper for each of the four clinical MRSA isolates (Supplementary Table S2). With MICs of 128, 32, and 64 µg/ml, three of the isolates, MRSA-1, -3, and -4, displayed resistance to ampicillin, while MRSA-2 was sensitive to ampicillin under the chosen experimental conditions, although the clinical resistance profile had labeled it as ampicillin resistant.

**Figure 5:**
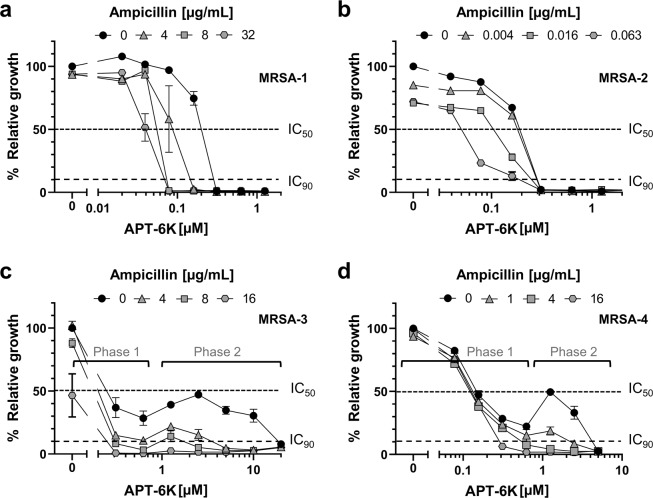
APT-6K restores ampicillin activity to synergistically inhibit ampicillin resistant MRSA. APT-6K (+50 µM Cu) and ampicillin were titrated against each other and tested against the four characterized clinical MRSA isolates. The ability of APT-6K to restore ampicillin activity allows the compound/drug combination to shift the IC_50_ and/or IC_90_ of APT-6K sensitive isolates (**a**) MRSA-1 or (**b**) MRSA-2 to even lower concentrations. For the APT-6K tolerant (**c**) MRSA-3 and (**d**) MRSA-4 isolates addition of physiologically relevant ampicillin concentrations overcame APT-6K tolerance and allowed APT-6K to suppress MRSA growth below the IC_90_ threshold. The utilized ampicillin concentrations are indicated and focus on the physiological relevant 1–8 µM range that can be accomplished in the serum of patients. Other drug concentrations are not shown for visual clarity. All values were normalized to the untreated controls of each series.

Guided by these concentration data, APT-6K and ampicillin were then titrated against each other in microplate assays and *S. aureus* growth was measured using optical density after overnight growth. For each of the MRSA isolates, the results provided evidence that the two drugs interacted and APT-6K restored the antibacterial effect of ampicillin in the otherwise resistant strains. The specific nature of the APT-6K/ampicillin interaction effects varied with observed differences in the ampicillin resistance and APT-6K tolerance profiles of the clinical isolates (Fig. 5).

For the APT-6K sensitive/ampicillin resistant MRSA-1 isolate, this is immediately visible by the ampicillin induced shift of the APT-6K IC_50_/IC_90_ values to lower concentrations (Fig. 5a). IC_50_/IC_90_ value reductions could be accomplished by as little as 8 µg/ml ampicillin which is within physiologically relevant ampicillin concentrations that can be readily achieved in patients. Similar observations were made with the APT-6K sensitive/ampicillin sensitive MRSA-2 isolate. Interestingly, addition of ampicillin, at much lower concentrations improved the APT-6K IC_50_ value, but not the IC_90_ value to lower concentrations (Fig. 5b). The most interesting results came from the two APT-6K tolerant/ampicillin resistant MRSA isolates (MRSA-3 and MRSA-4). For both, MRSA-3 and MRSA-4, which exhibit a bi-phasic response to APT-6K and for which APT-6K alone does not suppress bacterial growth below the IC_90_ threshold at a therapeutically relevant concentration, the addition of physiologically relevant ampicillin concentrations overcame APT-6K tolerance (Fig. 5c,d). For either isolate, ampicillin concentrations between 4–8 µg/ml in combination with 0.3–0.6 µM APT-6K were sufficient to suppress bacterial growth below the IC_90_ threshold. The ability of ampicillin to either lower the APT-6K IC_90_ (Fig. 5a) or to overcome bacterial APT-6K tolerance (Fig. 5c,d) in MRSA isolates at concentrations it would be inactive by itself (1–8 µg/ml) clearly demonstrates positive drug interactions between the two compounds/drugs, and this can only occur if APT-6K at least partially restores the antibacterial activity of ampicillin against these clinical MRSA isolates.

## Conclusions

Drug resistant infections in the US increased from 5% to 11% between 2002 and 2014, resulting in an annual ~2.2 billion dollar burden for the United States health care system^43^. To combat these bacteria, both new antibiotics as well as strategies to reduce resistance rates are urgently needed. In *S. aureus*, drug combinations to overcome pre-existing drug resistance with ß-lactam antibiotics have shown some promise in *in vitro* studies and in animal models, with human efficacy studies underway^39,44–47^. Our findings add APT-6K to the currently small list of compounds that can exert a direct antibacterial effect in the sub-micromolar range and can reverse ampicillin resistance at even lower concentrations. Also, APT-6K restored ampicillin activity to a concentration range (1–8 µg/ml) that is readily achievable in the serum of patients taking ampicillin^48,49^. Given the large body of literature that links not only copper-resistance, but more generally metal-resistance in bacteria with antibiotic resistance, the finding that some copper-drugs can overcome antibiotic resistance, exemplified by ampicillin resistance in this study, may actually not be surprising and should spur investigations into this phenomenon beyond MRSA (reviewed in: Poole 2017^38^). The exact mechanism of APT-6K/ampicillin synergy is subject of ongoing study. For APT-6K, the relatively favorable therapeutic index in the absence of mitochondrial toxicities is also a marked improvement over earlier CDIs such as GTSM. This low toxicity extends to primary human PBMCs, which after treatment do not exhibit noticeable changes in surface markers, activation markers, or inflammatory cytokines. As such, our findings add APT-6K to the growing list of effective metal-dependent compounds that form a new class of antibiotics and provide the first example of a CDI that can reverse antibiotic resistance to the classic ß-lactam antibiotic, ampicillin.

## Material and Methods

### Bacterial growth conditions, cell culture, compounds, and metals

*S. aureus* strain Newman was verified by whole genome sequencing. The MRSA strains used in this study were obtained in a deidentified manner from UAB Laboratory Medicine after confirmation of their drug resistance. The transposon mutants and the parent JE2 were obtained from BEI as part of the Nebraska Transposon Mutant Library. Bacteria were inoculated from stocks stored at −80 °C into Mueller Hinton media (Oxoid LTD) and incubated overnight at 37 °C with shaking at 180 rpm. THP-1 monocytes and PBMCs were cultured in Roswell Park Memorial Institute (RPMI) media (Corning) supplemented with 10% fetal bovine serum (FBS, Atlanta Biologicals, Inc.), glutamine, penicillin and streptomycin, and incubated at 37 °C in the presence of 5% CO_2._ APT-6K was purchased from ChemBridge Corp., reconstituted and aliquoted in DMSO at a 10 mM stock concentration, and stored at −80 °C. Ampicillin (Fisher Scientific) was reconstituted and aliquoted in ddH_2_O at 10 mg/mL and stored at −80 °C. Copper sulfate (Acros Organics), zinc sulfate (Fisher Scientific), manganese sulfate (Alfa Aesar), cobalt sulfate (Alfa Aesar), nickel sulfate (Acros Organics), and iron chloride (Acros Organics) were reconstituted and aliquoted in ddH_2_O at 100 mM and stored at 4 °C.

### Minimum inhibitory concentration (MIC) and metal specificity testing

Compound testing was performed in sterile 96-well plates. Compounds and the indicated metal salts were mixed in RPMI-1640 media without phenol red (Corning) that had a trace metal supplementation (RPMI-TM) to promote growth (3 µM EDTA, 50 µM MgCl_2_, 0.7 µM CaCl_2_, 80 µM NaMoO_4_, 168 µM CoCl_2_, 0.55 µM MnCl_2_, 0.7 µM ZnSO_4_, 2 µM FeSO_4_). Throughout the manuscript, metal concentrations refer to metal concentrations added in excess of the metals contained in this basic culture medium. Compound and antibiotic concentrations were titrated 2-fold from well to well. Bacteria were harvested from exponentially growing cultures that had been reinoculated from overnight cultures. The harvested bacteria were washed twice in RPMI-TM and then distributed to each well for a final OD of 0.005 (~5 × 10^6^ bacteria/mL). Within each plate, there were sterility controls and no compound controls that were used as blanks or as 100% growth, respectively. Plates were sealed with parafilm to reduce evaporation and kept at 37 °C with 5% CO_2_ for 18–24 hours, after which each well was resuspended and OD_600_ readings were taken on a plate reader (Cytation 3 or Synergy 2, BioTek). MIC was defined as the lowest concentration at which growth was reduced by at least 90% in comparison to the untreated controls.

### Time to death assay and bactericidal activity

Time to death and bactericidal activity assays were setup identically to that of the MIC determination assays. At the indicated time points or at the end of incubation (18–20 hours), the wells were resuspended and 5 µL of their contents were directly spotted onto MH agar plates and incubated overnight at 37 °C.

### Quantification of intracellular ATP concentrations

To determine the effect of the tested compounds on energy production, we determined ATP concentrations as a surrogate marker. APT-6K in the presence of 50 µM copper and CCCP were titrated on *S. aureus* strain Newman seeded at an OD of 0.005 in RPMI-TM medium and then incubated for 1 hour at 37 °C. At this time, the BacTiter-Glo assay (Promega) was used to measure the ATP content of treated bacteria. Luminescence was read on a Cytation 3 spectrophotometer.

### Testing of drug synergies

Microplate assays were performed in 96-well plates to determine the effects of copper or antibiotics when combined with APT-6K. The titrations of each test compound or metal ion were prepared in separate plates. The first plate had the test compound titrated across the plate (from column to column), while the second plate had the test compound or metal titrated 2-fold down the plate (from row to row). Volume from plate two was then transferred into plate one. Bacteria were washed twice and added to each well for a final assay OD of 0.005 (~5 × 10^6^ bacteria/mL). Each microplate assay was done in triplicate, and each plate had sterility controls for blanking and controls without compound for normalization. Plates were incubated for 18–24 hours at 37 °C with 5% CO_2_. After incubation, plates were resuspended and OD_600_ readings were taken on a plate reader.

### Toxicity of APT-6K on human cells

Toxicity of APT-6K on the monocytic THP-1 cells or peripheral blood mononuclear cells (PBMCs) isolated from buffy coats obtained from the Red Cross was assayed in the presence and absence of copper. Compounds were titrated and diluted in similar manner as described for *S. aureus*, but RPMI 1640 complemented with 10% FBS was used. After 24 hours of treatment, resazurin (Sigma-Aldrich) was added for a final concentration of 2.5 µg/mL. The viability status of the culture was analyzed by resazurin conversion resulting in fluorescence that was measured at excitation/emission: 530 nm/590 nm.

### Flow cytometric analysis and reagents

LIVE/DEAD Fixable Aqua Stain (Invitrogen), excited by violet 405 nm laser and detected by 512 nm emission channel, was used to determine the percentage of dead cells. The T cell phenotype was determined by staining with mAbs: CD3-APC efluor780 (SK7, eBiosciences), CD4-BV786 (SK3, Biolegend), CD8-V500 (RPA-T8, BD Biosciences), and CD28-BV711 (CD28.2, BD Biosciences). Activation status was determined using mAbs: PD-1-PE-eFlour610 (J105, eBioscience), CD38-BUV737 (HB7, BD Biosciences), CD69-FITC (FN50, BD Biosciences), and CD25-BUV395 (2A3, BD Biosciences). Cytolytic profile was determined using mAbs: TNF-α-APC (6401.1111, BD Biosciences), Granzyme B-BV421 (GB11; BD Biosciences), and IFN-γ-PE-cy7 (B27, BD Biosciences). Monoclonal Ab CD14 BUV805 (M5E2, BD Biosciences, Franklin Lake, NJ) was used to exclude monocytes from analysis. Intracellular staining (ICS) was performed using the BD Cytofix/Cytoperm kit according to the manufacturer’s protocols. All antibody-stained cells were fixed in 1% formaldehyde (Sigma) prior to sample acquisition on a Symphony flow cytometer (BD Biosciences). Gates for flow cytometric acquisition and analyses were based on “fluorescence-minus-one” (FMO) controls and single stain compensation controls.

### Mitochondrial toxicity

Mitochondrial toxicity was measured as a comparison of ATP levels and membrane integrity, using the Mitochondrial ToxGlo system (Promega). The assay was carried out as described by the manufacturer in a white 384 well-plate. THP-1 cells were grown in standard RPMI 1640 medium, then passaged for one day prior to the experiment in glucose-free RPMI supplemented with 0.2% galactose to better visualize mitochondrial inhibition. The assay itself was conducted using FBS-free and galactose supplemented RPMI. Digitonin and CCCP, as recommended by the manufacturer, were used as cytotoxicity and mitochondrial toxicity controls, respectively. Cytotoxicity was normalized using the highest digitonin concentration as 0% viability, and the no treatment control as 100% viability.

### PCR and gel electrophoresis

Colony PCR was used to amplify the genes of interest. Briefly, a colony of the test strain was diluted in water and then added as the DNA source for the PCR using a Taq polymerase master mix (NEB). The PCR conditions used were based on the manufacture’s recommendations with an annealing temperature of 62 °C for 30 seconds and an extension time of 3 mins for 30 cycles.

Primers were designed to amplify the full gene and the primer sequences are provided in Supplementary Table 3. The PCR products were visualized after separation on a 1% agarose gel. The expected product sizes were 2791 bp for *copA*, 566 bp for *copZ*, 2729 bp for *copX*, and 1263 bp for *copL*.

### Data analysis and chemical structures

Each experiment is representative of at least two independent experiments and contains at least 3 technical replicates. Error bars represent standard deviation of technical replicates unless stated otherwise. Data were analyzed and graphed using Excel (Microsoft), GraphPad Prism 8 (GraphPad), FlowJo (Treestar), and PowerPoint (Microsoft). Primers were designed using SnapGene (GSL Biotech LLC). Marvin Sketch was used for drawing and displaying chemical structures (MarvinSketch 6.1.4, 2013, ChemAxon (http://www.chemaxon.com)).

## Supplementary information

Supplementary Information.
