# Supplementary material for: A copper-dependent compound restores ampicillin sensitivity in multidrug-resistant Staphylococcus aureus
Source: Sci Rep. 2020 Jun 2;10:8955. doi: 10.1038/s41598-020-65978-y (PMC7265353; doi:10.1038/s41598-020-65978-y)
Supplement: Supplementary file 1 — Supplementary Information. [file 41598_2020_65978_MOESM1_ESM.pdf]

Supplementary Figures and Tables

Title: "A copper-dependent compound restores ampicillin sensitivity in multidrug-resistant *Staphylococcus aureus"*

Authors: Cameron L. Crawford[1], Alex G. Dalecki[1], Mildred D. Perez[1], Kaitlyn Schaaf[1], Frank Wolschendorf[1], and Olaf Kutsch[1]

[1]Department of Medicine, University of Alabama at Birmingham, Birmingham, AL, USA

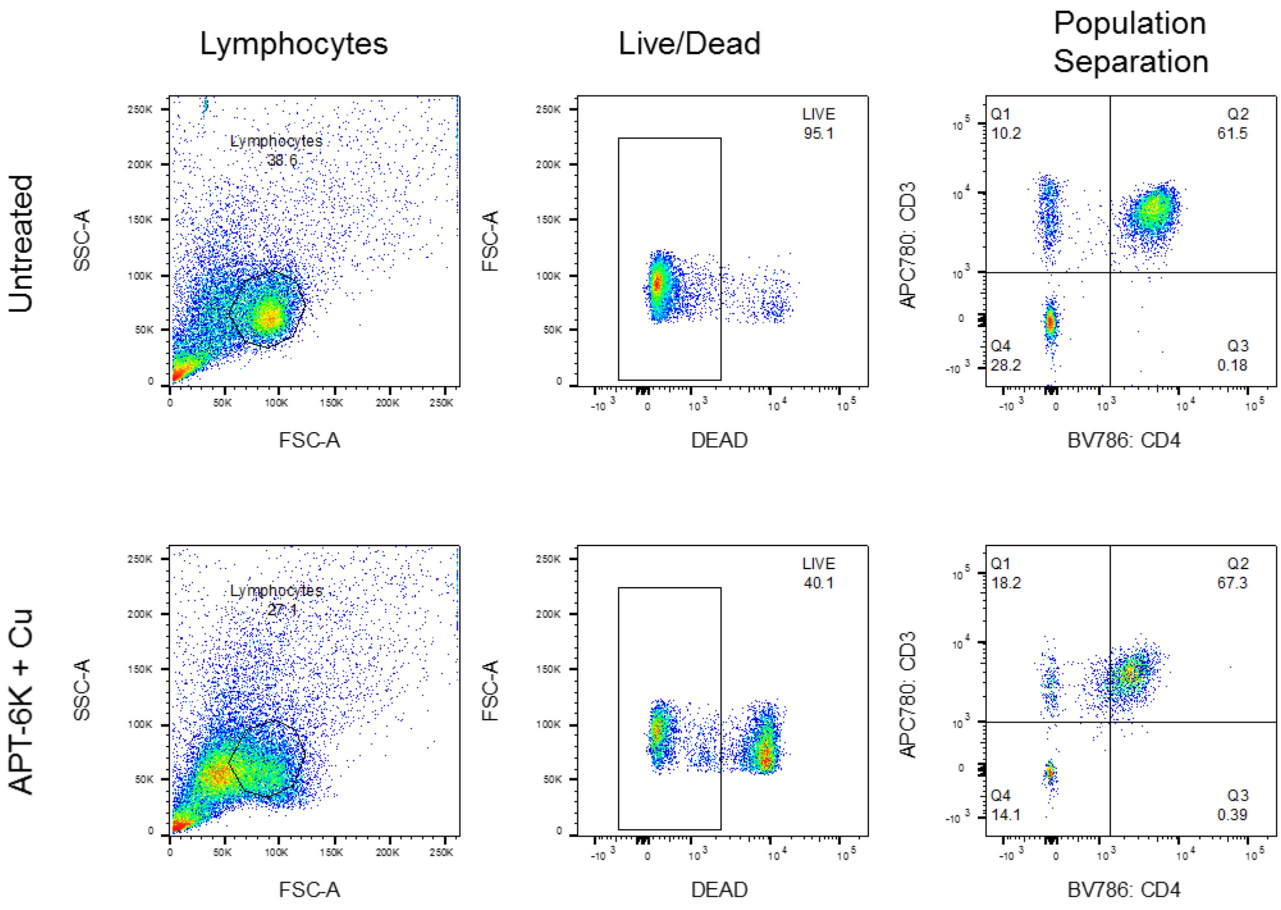

Supplementary Figure 1: Flow cytometric analysis of compound toxicities. Flow cytometric analysis was used to detect APT-6K cytotoxicity effects on peripheral blood mononuclear cells. The flow cytometric analysis dot plots are representative for data generated using PBMC samples from 6 healthy human individuals. Forward scatter (cell size)/Side scatter (granularity) provide primary information on cell health. Left: Cells within the gate meet the expected size and granularity of lymphocytes. Middle: A live/dead stain exclusively stain dead cells. Right: Stain resolving the PBMC population for CD3 and CD4 expression. Upper panels: untreated control cells. Lower panels: cells treated with a toxic concentration of APT-6K.

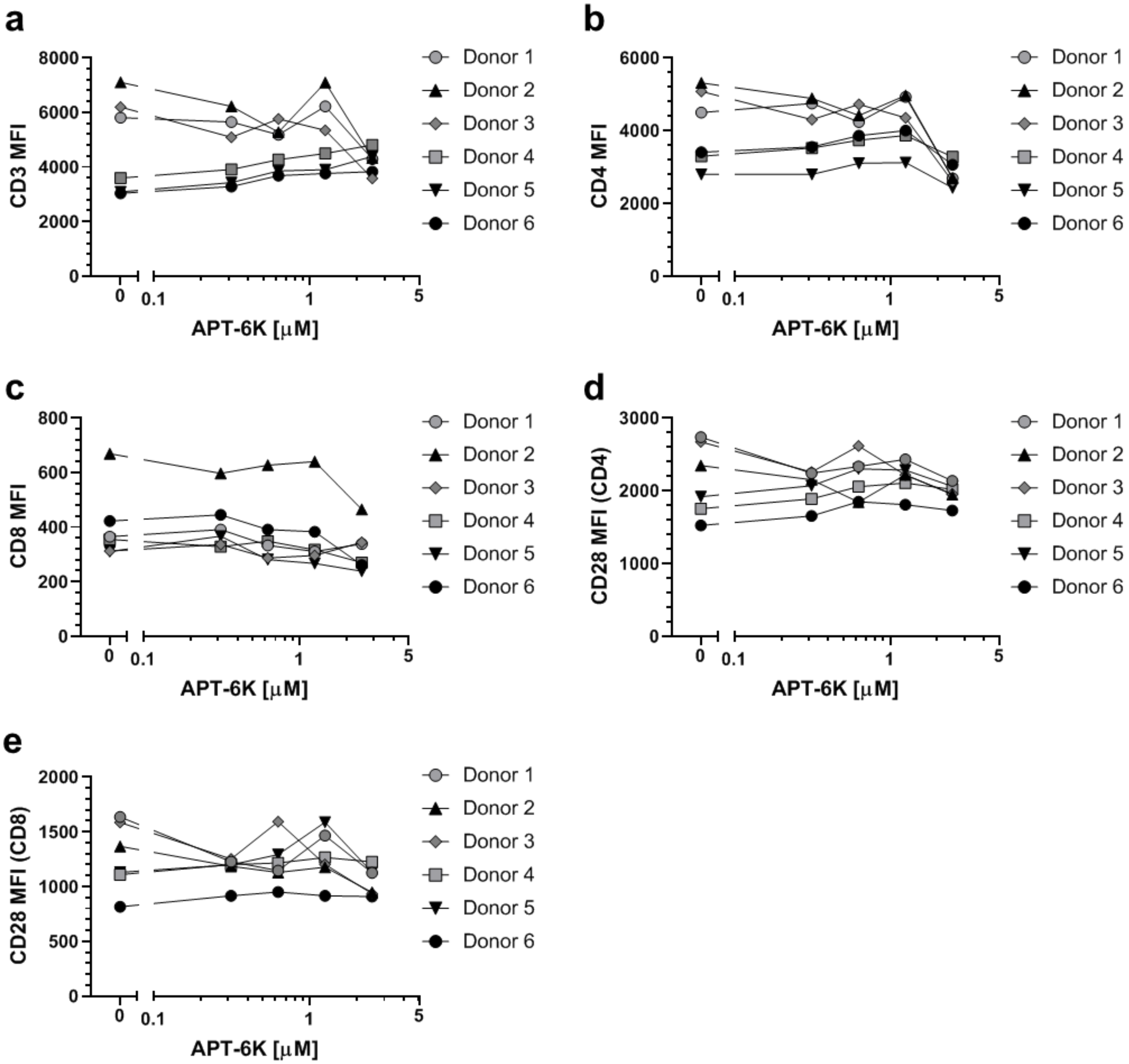

Supplementary Figure 2: Mean fluorescence intensity of surface markers found on T cells treated with APT-6K. PBMCs from six donors were treated with increasing concentrations of APT-6K and 50 µM copper for 24 hours and then stained for (**a**) CD3 expression, (**b**) CD4 expression, (**c**) CD8 expression, (**d**) CD28 expression on CD4 T cells, and (**e**) CD28 on CD8 T cells**.** Data are presented as mean fluorescence intensity.

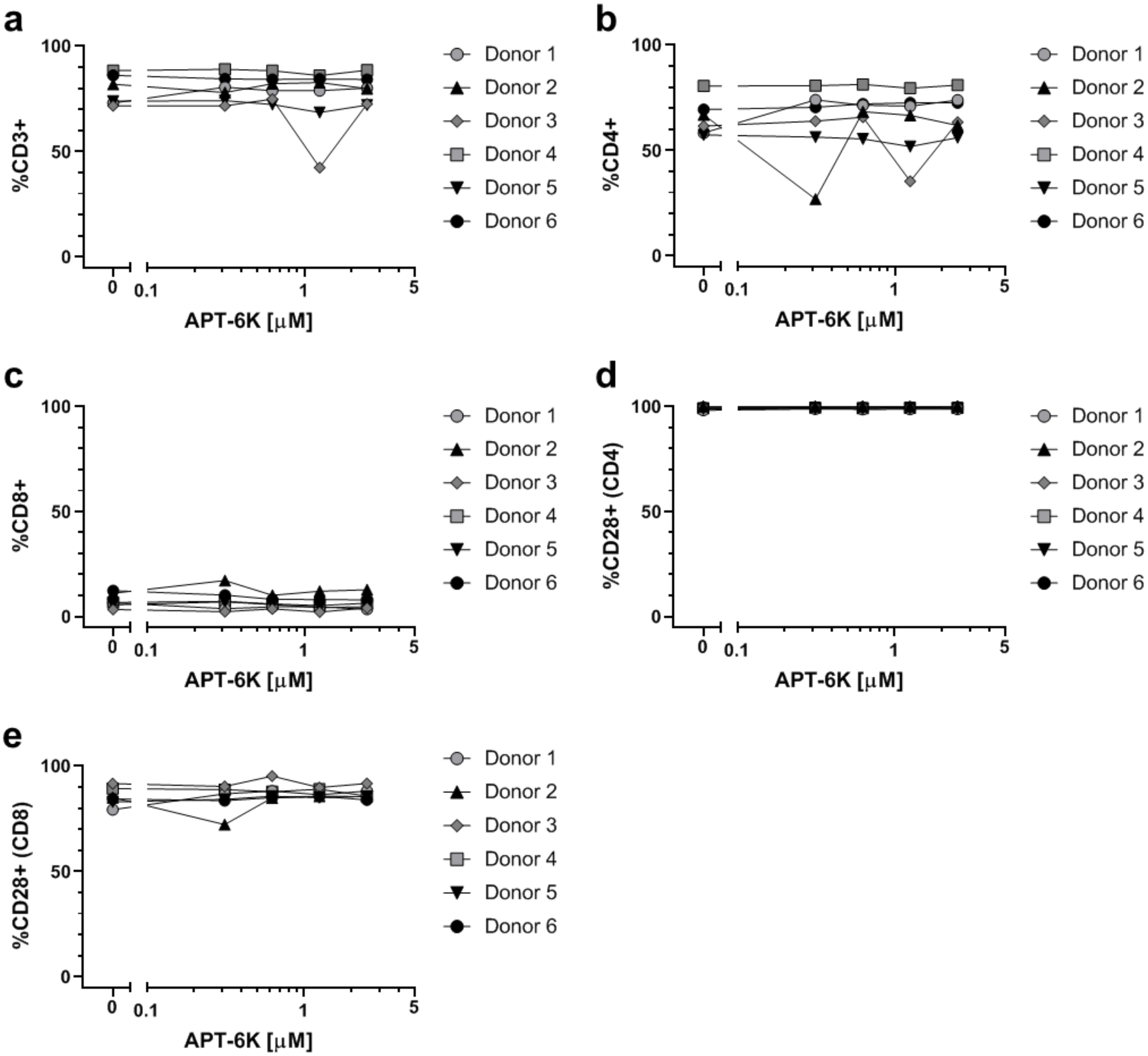

Supplementary Figure 3: Effect of APT-6K treatment on the composition of the T cell population. PBMCs from six donors were treated with increasing concentrations of APT-6K and 50 µM copper for 24 hours and then stained for (**a**) CD3 expression, (**b**) CD4 expression, (**c**) CD8 expression, (**d**) CD28 expression on CD4 T cells, and (**e**) CD28 on CD8 T cells**.** Data are presented as percentage of cells carrying the respective marker.

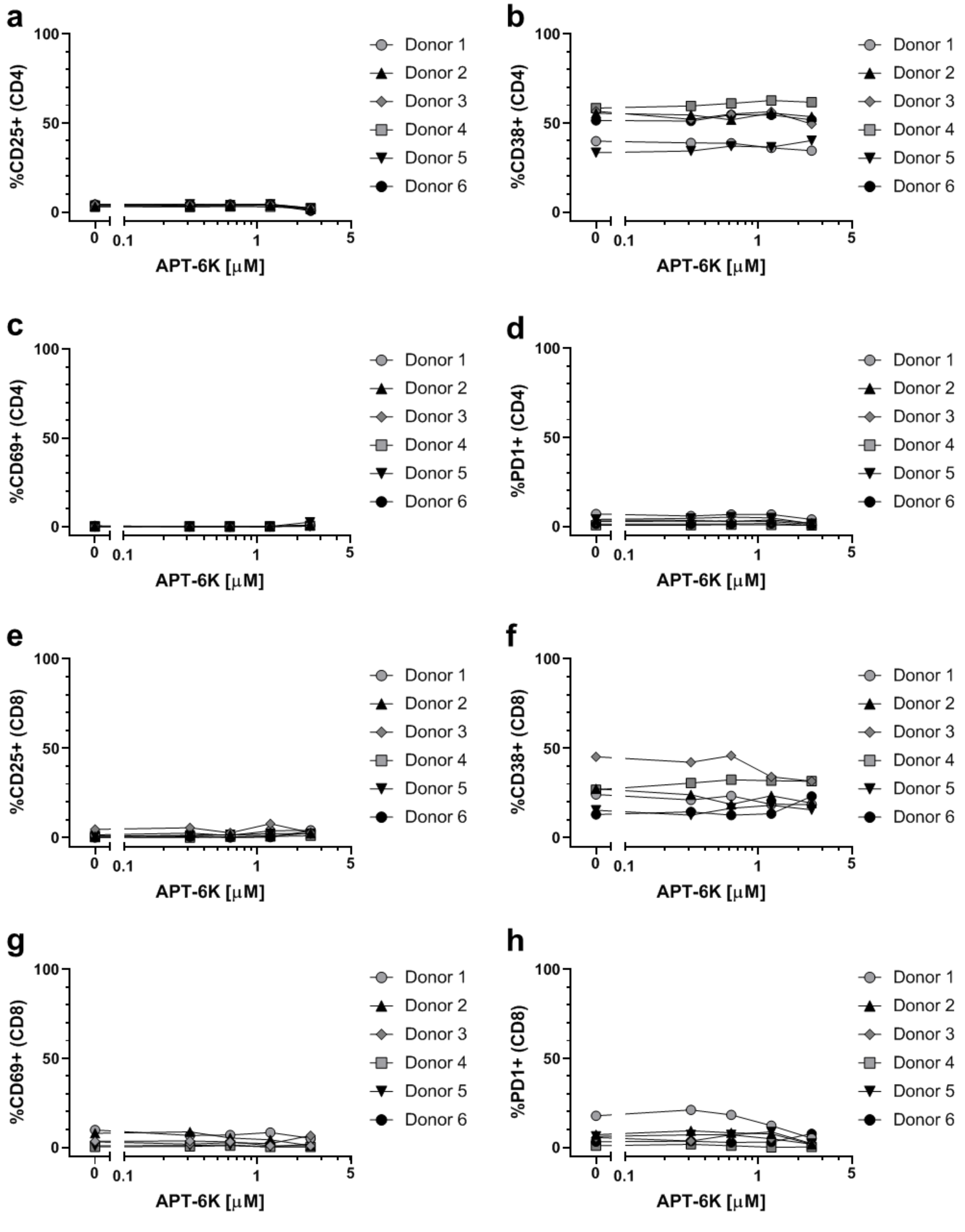

Supplementary Figure 4: Effect of APT-6K treatment on the expression of T cell activation markers. PBMCs from six donors were treated for 24 hours with APT-6K with copper and then stained for the expression of (**a**) CD25 on CD4 T cells, (**b**) CD38 on CD4 T cells, (**c**) CD69 on CD4 T cells, (**d**) PD1 on CD4 T cells, (e) CD25 on CD8 T cells, (**f**) CD38 on CD8 T cells, (**g**) CD69 on CD8 T cells, and (**h**) PD1 on CD8 T cells. Data are presented as percentage of cells carrying the respective marker.

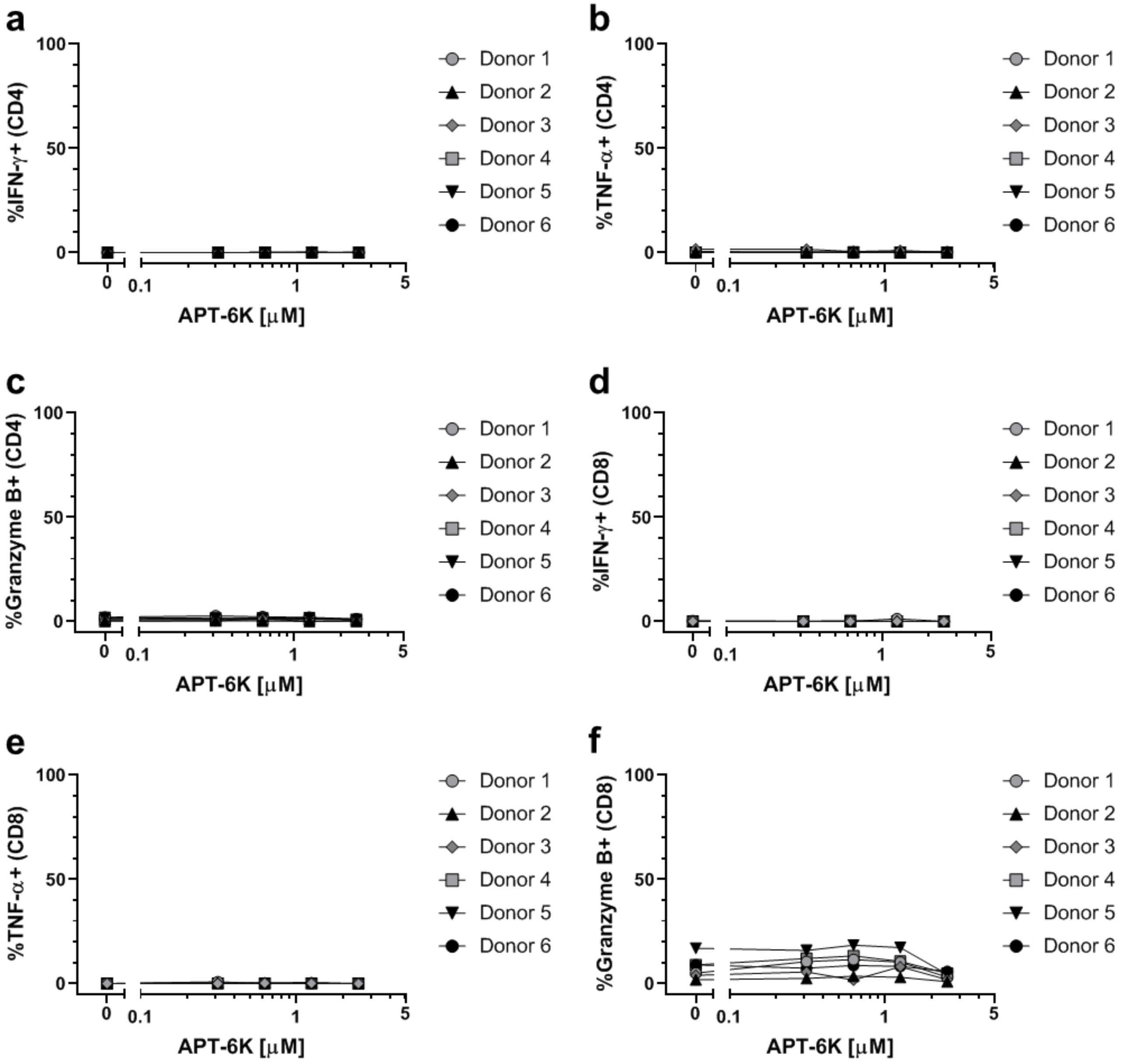

Supplementary Figure 5: Absence of induction of inflammatory cytokine by APT-6K. PBMCs from six donors were treated for 24 hours with APT-6K and copper and then stained with antibodies against (**a**) IFN-γ in CD4 T cells, (**b**) TNF-α in CD4 T cells, (**c**) Granzyme B in CD4 T cells, (**d**) IFN-γ in CD8 T cells, (**e**) TNF-α in CD8 T cells, (**f**) Granzyme B in CD4 T cells. Data are presented as percentage of cells carrying the respective marker.

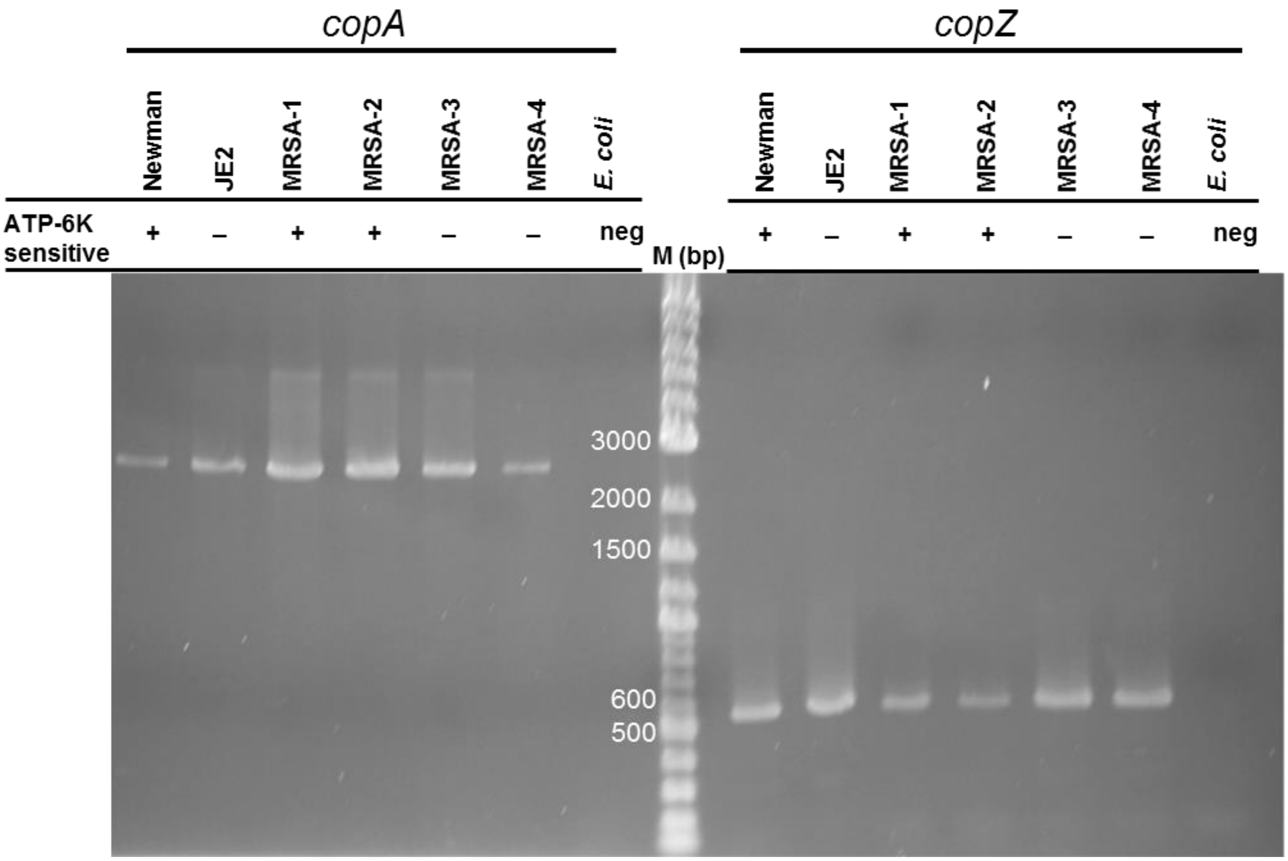

Supplementary Figure 6: All tested *S. aureus* strains carry the copper resistance genes *copA* and *copZ.* Presentation of the unedited PCR gels shown in Figure 4. Primers for the full length genes of *copA* and *copZ* were used in colony PCRs to amplify the target genes from Newman, JE2, MRSA-1, MRSA-2, MRSA-3, MRSA-4, and *E. coli* (negative control).

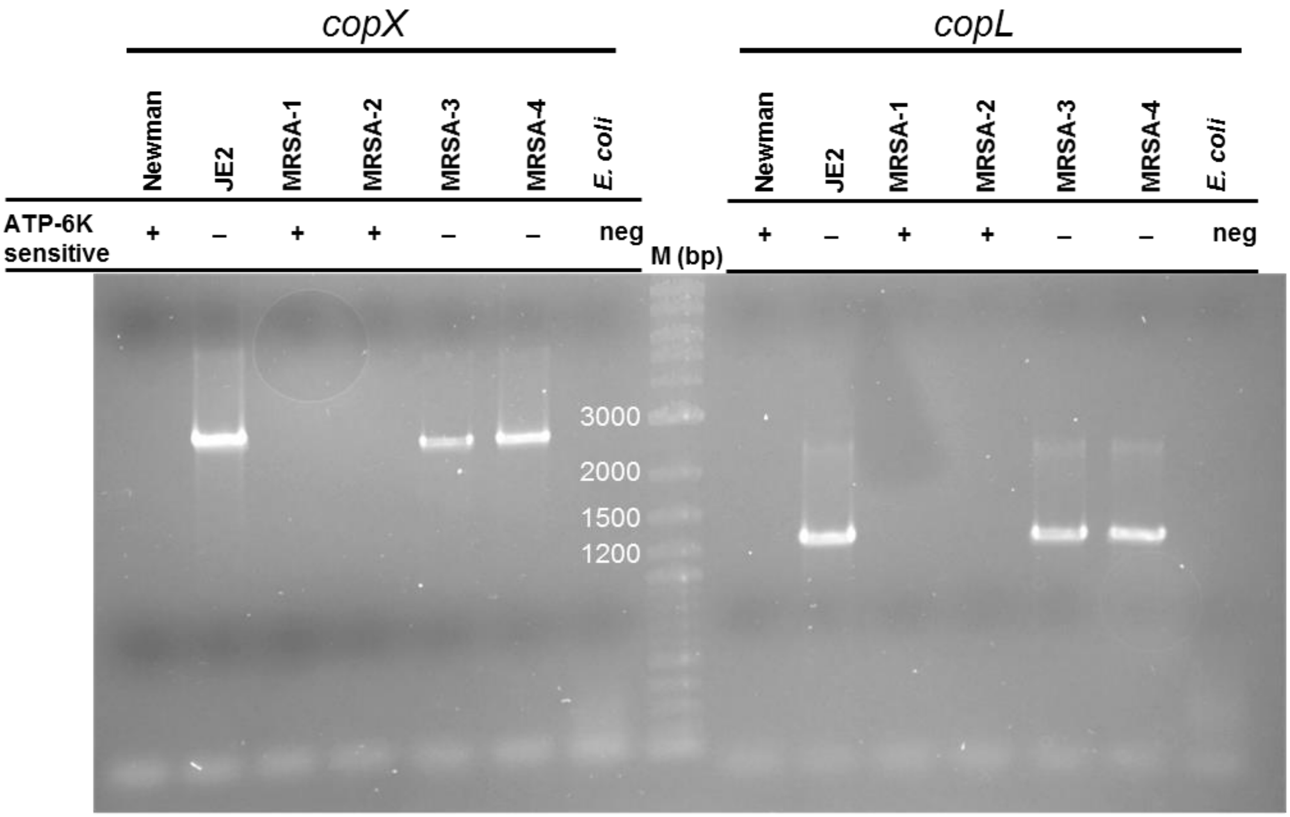

Supplementary Figure 7: All tested *S. aureus* strains contain the copper resistant genes *copA* and *copZ.* Presentation of the unedited PCR gels shown in Figure 4. Primers for the full length genes of *copX* and *copL* were used in colony PCRs to amplify the target genes from Newman, JE2, MRSA-1, MRSA-2, MRSA-3, MRSA-4, and *E. coli* (negative control).

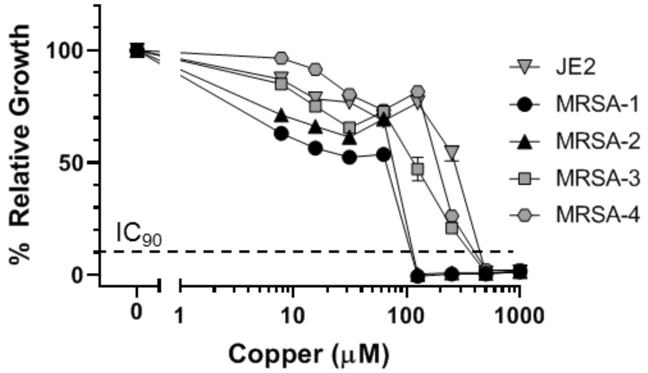

Supplementary Figure 8: Copper sensitivity of MRSA strains. JE2, and the four MRSA strains were treated with dilutions of copper. All normalized values have been normalized to the untreated controls of each series.

Table S1: Clinical resistance profiles of selected MRSA isolates

	Ampicillin	Ciprofloxacin	Clindamycin	Daptomycin	Erythromycin	Gentamycin	Levofloxacin	Linezolid	Sulfamethaxole	Tetracycline	Vancomycin
**MRSA-1**	R	S	R	S	R	S	S	S	S	R	S
**MRSA-2**	R	R	R	S	R	S	R	S	S	R	S
**MRSA-3**	R	S	S	S	R	S	S	S	S	S	S
**MRSA-4**	R	S	S	S	R	S	S	S	S	S	S

Table S2: APT-6K and ampicillin resistance profiles of selected MRSA isolates

	Ampicillin [µg/ml]	APT-6K [µM]*
**MRSA-1**	128	0.31
**MRSA-2**	1	0.31
**MRSA-3**	32	20
**MRSA-4**	64	5

* +50µM Cu

Table S3: Primers used in this study.

Primer	Sequence	Tm (°C)
*copA*_full_F	CACGAGTCGTCAGTTGTCAG	62.4
*copA*_full_R	CTCAGAATCACCAACGAATC	58.4
*copZ*_full_F	AGTGGCTCTAAAAGGAGGTG	60.4
*copZ*_full_R	TGGTGTGATTAAAGGTGCAG	58.4
*copL*_full_F	AGTGATGTCCACGCACAAC	60.2
*copL*_full_R	TCCACCATTTAACACCCTCC	60.4
*copX*_full_F	CAATTTGCGTTTCTAGGCATAC	58.9
*copX*_full_R	TTAATCTCTTCGTTTACGACCC	58.9
